# Sedentary Behaviour, Visceral Fat Accumulation and Cardiometabolic Risk in Adults: A 6-Year Longitudinal Study from the Quebec Family Study

**DOI:** 10.1371/journal.pone.0054225

**Published:** 2013-01-09

**Authors:** Travis J. Saunders, Mark S. Tremblay, Jean-Pierre Després, Claude Bouchard, Angelo Tremblay, Jean-Philippe Chaput

**Affiliations:** 1 Healthy Active Living and Obesity Research Group, Children's Hospital of Eastern Ontario Research Institute, Ottawa, Ontario, Canada; 2 School of Human Kinetics, Faculty of Health Sciences, University of Ottawa, Ottawa, Ontario, Canada; 3 Department of Pediatrics, Faculty of Medicine, University of Ottawa, Ottawa, Ontario, Canada; 4 Department of Cardiology, Centre de Recherche de l′Institut Universitaire de Cardiologie et de Pneumologie de Québec, Quebec City, Quebec, Canada; 5 Department of Kinesiology, Faculty of Medicine, Laval University, Quebec City, Quebec, Canada; 6 Human Genomics Laboratory, Pennington Biomedical Research Center, Baton Rouge, Louisiana, United States of America; McGill University, Canada

## Abstract

**Background:**

Sedentary behaviour has recently emerged as a unique risk factor for chronic disease morbidity and mortality. One factor that may explain this relationship is visceral adiposity, which is prospectively associated with increased cardiometabolic risk and mortality. The objective of the present study was to determine whether sedentary behaviour was associated with increased accumulation of visceral fat or other deleterious changes in cardiometabolic risk over a 6-year follow-up period among adult participants in the Quebec Family Study.

**Methods:**

The current study included 123 men and 153 women between the ages of 18 and 65. Total sedentary time and physical activity were assessed by self-report questionnaire. Cross-sectional areas of visceral and subcutaneous abdominal adipose tissue were assessed using computed tomography. Cardiometabolic biomarkers including fasting insulin, glucose, blood lipids, HOMA-Insulin Resistance, and oral glucose tolerance were also measured. All variables of interest were collected at both baseline and follow-up.

**Results:**

After adjustment for age, sex, baseline BMI, physical activity, energy intake, smoking, education, income and menopausal status, baseline sedentary behaviour was not associated with changes in visceral adiposity or any other marker of cardiometabolic risk. In the longitudinal model which adjusted for all studied covariates, every 15-minute increase in sedentary behaviour from baseline to follow-up was associated with a 0.13 cm increase in waist circumference (95% CI = 0.02, 0.25). However, there was no association between changes in sedentary behaviour and changes in visceral adiposity or other markers of cardiometabolic risk.

**Conclusion:**

These results suggest that neither baseline sedentary behaviour nor changes in sedentary behaviour are associated with longitudinal changes in visceral adiposity in adult men and women. With the exception of waist circumference, the present study did not find evidence of a relationship between sedentary behaviour and any marker of cardiometabolic risk in this population.

## Introduction

Sedentary behaviour (e.g. sitting, reclining) has recently emerged as a unique risk factor for chronic disease [1], [2] and is consistently associated with increased risk of both obesity and mortality [3]–[6]. Excess sedentary time has also been associated with increased accumulation of central adiposity and other markers of cardiometabolic risk [7], [8]. For example, Wijndaele and colleagues reported that increases in television (TV) viewing during a 5-year follow-up period were associated with significant increases in waist circumference in both men and women, and increases in blood pressure and clustered cardiometabolic risk among women [8].

One factor that may link sedentary behaviour with increased morbidity and mortality is the accumulation of visceral adipose tissue, which is prospectively associated with mortality and increased cardiometabolic risk [9]–[11]. Despite the hypothesized link between high levels of sedentary behaviour and both obesity and central adiposity, the association between sedentary behaviour and the accumulation of visceral adipose tissue remains largely unexamined. To our knowledge, only one cross-sectional study has examined this question, and reported no association between sedentary behaviour and visceral fat levels in physically inactive adults [12].

A longitudinal study of the association between sedentary behaviour and the accumulation of visceral fat could therefore make an important contribution to our understanding of the relationship between sedentary behaviour and chronic disease morbidity and mortality. The objective of the present study was to determine whether sedentary behaviour was associated with increased accumulation of visceral fat or other deleterious changes in cardiometabolic risk among adult participants in the Quebec Family Study.

## Materials and Methods

### Ethics Statement

All participants provided written informed consent to participate in the study. The project followed guidelines of the Medical Research Council of Canada, and was approved by the Medical Ethics Committee of Laval University.

### Subjects

The Quebec Family Study was initiated at Laval University in 1978. The primary goal of this project was to investigate the role of genetics in the development of obesity and related cardiovascular risk factors. A total of 1650 individuals from 375 families were recruited and assessed in Phase 1 of the study (1978 to 1981). In this initial phase recruitment was conducted irrespective of body weight, resulting in a cohort with body mass index (BMI), ranging from 13.8 to 64.9 kg/m^2^. An additional 123 families with at least 1 parent and 1 offspring with a BMI of 32 or higher were added to the study in Phase 2 (1989–1994) and 3 (1995–2001) of the study, while also retesting 100 families from Phase 1. Families were recruited through the media and were all French Canadians from the greater Québec City area. From the sample of 223 white nuclear families (totaling 951 subjects involved in Phases 1, 2, and 3), 147 men and 169 women were eligible for longitudinal analyses between Phase 2 and 3. Longitudinal analyses were not possible with Phase 1 as assessments differed at this time point from those employed in Phases 2 and 3. Additional details about the Quebec Family Study have been previously published [13].

Baseline in the current study corresponded to Phase 2, and the mean duration of follow-up between Phase 2 and 3 was 6.0 (SD 1.0) years. The following exclusion criteria were applied: (i) aged less than 18 years or greater than 64 years (13 men and 9 women excluded); (ii) diabetic, defined as use of insulin or a hypoglycemic agent, a fasting plasma glucose level of ≥7.0 mmol/L, or a 2-hour postload plasma glucose level of ≥11.1 mmol/L (7 men and 3 women excluded) and (iii) missing data for sedentary behaviour (4 men and 4 women excluded). The final number of eligible participants within the longitudinal sample was 286 individuals (123 men and 153 women) (see Tables 1 and 2).

**Table 1:** Baseline subject characteristics across tertiles of baseline sedentary behaviour in males.

	n (L/M/H)	Low	Medium	High
Age (years)	39/33/51	39 (15)	39 (13)	39 (16)
Baseline sedentary time (min/day)	39/33/51	305 (62)	472 (50)	667 (89)
MVPA (min/day)	39/32/51	50 (60)	41 (47)	25 (36)
BMI (kg/m^2^)	39/33/51	25.8 (4.4)	26.5 (4.6)	26.4 (5.5)
Waist circumference (cm)	39/33/51	89.4 (12.6)	91.3 (12.1)	90.9 (15.6)
Body fat (%)	37/31/46	21.3 (7.5)	22.5 (7.4)	21.5 (8.6)
Visceral AT (cm^2^)	29/29/36	112.5 (61.1)	124.9 (67.0)	114.7 (85.5)
Subcutaneous AT (cm^2^)	29/29/36	208.2 (130.0)	235.5 (132.1)	191.6 (129.6)
Total abdominal AT (cm^2^)	29/29/36	320.7 (176.9)	360.4 (189.5)	306.3 (197.6)
Fasting glucose (mmol/L)	39/33/51	5.00 (0.59)	4.93 (0.57)	4.97 (0.52)
HOMA-IR Index	28/30/39	2.32 (1.72)	2.96 (2.77)	2.87 (2.46)
Glucose AUC (mmol/L)	30/28/37	1224 (244)	1126 (197)	1143 (253)
Insulin AUC (pmol/L)	28/28/36	69249 (67670)	65615 (43890)	73260 (50413)
Total cholesterol (mmol/L)	39/22/50	4.95 (0.86)	5.15 (0.90)	4.89 (1.02)
HDL-cholesterol (mmol/L)	39/33/50	1.16 (0.30)	1.13 (0.29)	1.08 (0.25)
LDL-cholesterol (mmol/L)	38/32/50	3.17 (0.72)	3.25 (0.73)	3.13 (0.88)
Triglycerides (mmol/L)	39/33/50	1.49 (0.90)	1.77 (1.18)	1.56 (0.76)
Energy intake (kcal/day)	39/32/51	2910 (598)	2714 (715)	2672 (803)
Total family income in Canadian dollars (n (%))				
<10,000$		0 (0)	0 (0)	0 (0)
10,000–29,000$		1 (2.6)	0 (0)	0 (0)
30,000–49,000$		13 (34.2)	13 (37.1)	8 (17.0)
50,000–69,000$		13 (34.2)	9 (25.7)	13 (27.7)
70,000$+		11 (28.95)	13 (37.1)	26 (55.3)
Education level				
High School		20 (48.8)	9 (25.7)	10 (21.7)
College*		14 (34.2)	12 (34.3)	21 (45.7)
University		7 (17.1)	14 (40.0)	15 (32.6)

Data are expressed as mean (SD) unless otherwise specified.

L = low sedentary behaviour at baseline; M = medium sedentary behaviour at baseline; H = high sedentary behaviour at baseline; MVPA, moderate-to-vigorous physical activity; BMI, body mass index; AT, adipose tissue; HOMA-IR, homeostasis model assessment of insulin resistance; AUC, area under-the-curve.

* In Québec, there is a level of education generally lasting 2 to 3 years between high school and university termed CEGEP (*Collège d'Enseignement Général et Professionnel*), an acronym that does not have any translation in English.

**Table 2:** Baseline subject characteristics across tertiles of baseline sedentary behaviour in females.

	n (L/M/H)	Low	Medium	High
Age (years)	54/58/41	41 (12)	42 (15)	32 (13)
Baseline sedentary time (min/day)	54/58/41	305 (70)	463 (41)	620 (69)
MVPA (min/day)	53/57/30	21 (26)	10 (15)	14 (16)
BMI (kg/m^2^)	54/58/41	25.2 (6.0)	25.7 (6.3)	24.5 (4.7)
Waist circumference (cm)	54/58/41	77.9 (14.5)	79.0 (13.8)	76.8 (11.9)
Body fat (%)	45/50/40	30.8 (10.1)	31.2 (9.0)	28.4 (8.3)
Visceral AT (cm^2^)	41/42/29	87.7 (61.8)	101.2 (55.9)	64.4 (44.0)
Subcutaneous AT (cm^2^)	41/42/29	282.8 (154.8)	312.5 (162.6)	268.1 (147.4)
Total abdominal AT (cm^2^)	41/42/29	370.5 (202.2)	413.7 (200.9)	332.5 (180.6)
Fasting glucose (mmol/L)	54/56/40	4.66 (0.51)	4.81 (0.52)	4.69 (0.40)
HOMA-IR Index	44/45/32	2.29 (1.96)	2.27 (2.00)	1.94 (1.27)
Glucose AUC (mmol/L)	40/41/29	1075 (209)	1162 (257)	1101 (186)
Insulin AUC (pmol/L)	39/41/29	66290 (46483)	79134 (74763)	61007 (42899)
Total cholesterol (mmol/L)	5457/39	5.10 (1.01)	5.16 (1.03)	5.01 (2.00)
HDL-cholesterol (mmol/L)	54/5739	1.40 (0.36)	1.37 (0.32)	1.32 (0.37)
LDL-cholesterol (mmol/L)	54/5738	3.13 (0.86)	3.18 (0.86)	2.84 (1.00)
Triglycerides (mmol/L)	54/57/39	1.28 (0.59)	1.39 (0.62)	2.08 (5.11)
Energy intake (kcal/day)	54/56/41	1877 (381)	1869 (398)	2096 (434)
Total family income in Canadian dollars (n (%))				
<10,000$		1 (1.8)	2 (3.6)	1 (2.8)
10,000–29,000$		1 (1.8)	0 (0)	0 (0)
30,000–49,000$		25 (45.5)	16 (29.1)	6 (16.7)
50,000–69,000$		13 (23.6)	14 (25.5)	10 (27.8)
70,000$+		15 (27.3)	23 (41.8)	19 (52.8)
Education level				
High School		29 (50.9)	30 (50.9)	10 (27.0)
College*		15 (26.3)	21 (35.6)	16 (43.2)
University		13 (22.8)	8 (13.6)	11 (29.7)
Menopausal status				
In menopause		13 (36.1)	15 (46.9)	3 (30)
Not in menopause		23 (63.9)	17 (53.1)	7 (70)

Data are expressed as mean (SD) unless otherwise specified.

L = low sedentary behaviour at baseline; M = medium sedentary behaviour at baseline; H = high sedentary behaviour at baseline; MVPA, moderate-to-vigorous physical activity; BMI, body mass index; AT, adipose tissue; HOMA-IR, homeostasis model assessment of insulin resistance; AUC, area under-the-curve.

* In Québec, there is a level of education generally lasting 2 to 3 years between high school and university termed CEGEP (*Collège d'Enseignement Général et Professionnel*), an acronym that does not have any translation in English.

### Sedentary Behaviour and Physical Activity

Sedentary behaviour and physical activity were estimated using a physical activity record [14]. Subjects had to complete a physical activity diary for 3 days, including 2 weekdays and 1 weekend day, with each day being divided into 96 periods of 15 minutes each. Subjects were asked to code the main activity performed during each 15-minute period using a scale from 1 to 9, ranging from sleeping (category 1) to intense manual work (category 9). Time spent in categories 6 to 9 was used to calculate moderate-to-vigorous physical activity (MVPA) [14]. Sedentary behaviour was calculated as the sum of time identified as being in category 2 (“Sitting: eating, listening, writing, etc”). Time spent in category 1 (“sleeping, resting in bed”) was not included in the sedentary behaviour category as sedentary behaviour refers only to waking behaviours [2]. The reliability and validity of the record have been previously reported [14]. These measurements were performed both at baseline and after 6 years.

### Assessment of Abdominal Fat by Computed Tomography (CT)

Cross-sectional abdominal adipose tissue areas were assessed by CT using a Siemens Somatom DRH scanner (Erlanger, Germany) as described in detail elsewhere [15]. Briefly, an abdominal scan was taken between the fourth and fifth lumbar vertebrae (L4–L5) with subjects lying in a supine position with arms stretched above the head. The position of the scan was determined using a scout radiograph of the abdomen. Total and visceral adipose tissue areas were delineated with a graph pen and then computed using an attenuation range of −190 to −30 Hounsfield units [16]. Visceral fat area was determined by drawing a line within the muscle wall surrounding the abdominal cavity. Abdominal subcutaneous fat area was obtained by computing the difference between total and visceral adipose tissue areas.

### Anthropometric and Body Composition Measurements

Height was measured to the nearest 0.1 cm using a standard stadiometer, and body weight was measured to the nearest 0.1 kg using a digital panel indicator scale (Beckman Industrial Ltd, Model 610/612, Scotland, UK). BMI was calculated as body weight divided by height squared (kg/m^2^). Waist circumference was measured at the line between the lower border of the last rib and the upper border of the iliac crest. All anthropometric measurements were performed according to standardized procedures recommended at The Airlie Conference [17]. Body density was obtained from the mean of 6 valid measurements derived from underwater weighing [18]. The helium dilution method of Meneely and Kaltreider [19] was used to determine the pulmonary residual volume before immersion in the hydrostatic tank,. Total body fat percentage was determined from body density with the equation of Siri [20]. Body fat mass was estimated from body weight and the percentage of body fat. These measurements were performed in the same way at both baseline and after 6 years.

### Cardiometabolic Risk Factors

Total cholesterol and triglyceride concentrations were determined by use of commercial enzymatic-based methods, as described elsewhere [21]. HDL-cholesterol concentrations were analyzed after precipitation of apolipoprotein B-containing lipoproteins with heparin and manganese chloride [22]. Glucose concentrations were measured enzymatically and serum insulin concentrations were measured by radioimmunoassay [23]. A 75 g oral glucose tolerance test (OGTT) was performed in the morning after a 12 h fast. The total areas under the curve during the OGTT for insulin and glucose were computed from the plasma levels determined at 15 min intervals during the first hour following the glucose ingestion and every 30 min for the subsequent 3 h, using the trapezoidal method as previously described [24]. Insulin sensitivity was estimated in the fasting state using the homeostasis model assessment for insulin resistance (HOMA-IR) [25].

### Energy Intake

Diet was evaluated with a 3-day food record, including 2 weekdays and 1 weekend day, at baseline and year 6. Participants were shown how to complete this record by a dietician who provided instruction about measuring the quantities of ingested foods [26]. Mean daily energy intake was estimated by a dietician using a computerized version of the Canadian Nutrient File [27].

### Measurement of Covariates

Several covariates were measured via self-reported questionnaires. These include age, sex, smoking habits (nonsmoker or ex-smoker, light smoker [≤10 cigarettes per day], heavy smoker [>10 cigarettes per day]), highest educational level (high school, college [CEGEP for Quebec], university), total annual family income (categorized into 5 groups ranging from < $10,000 to $70,000 or more) and menopausal status.

### Statistical Analysis

Sample size calculations were performed to assess whether the present dataset was likely to provide sufficient power to detect a significant relationship between sedentary behaviour and longitudinal changes in our primary outcome of visceral adiposity, should one exist. Assuming that the fully adjusted model would account for roughly 25% of the variance in changes in visceral adiposity during the 6-year follow-up, and that sedentary behaviour would account for at least 3% of this variance, the current dataset of 206 participants with full data for our primary outcome provides more than 85% power to detect a significant association should one exist at an alpha level of 0.05.

To determine if men and women could be combined into one analysis, sex-by-sedentary behaviour interactions were assessed for all dependent variables. Significant interactions were detected for BMI and HDL-cholesterol, and thus analyses involving these variables are presented in men and women separately. There were no significant sex interactions for any other variables of interest, therefore all other analyses present men and women combined in order to maximize statistical power. Normality of distribution was assessed using the Shapiro-Wilk test and visual inspection. Moderate- and vigorous-intensity physical activity and BMI were both transformed using a log function. Regression analyses were performed to determine the univariate and multivariate associations between sedentary behaviour and 6-year changes in markers of cardiometabolic risk. Multivariate models were adjusted for age, sex, baseline BMI, energy intake, moderate- and vigorous-intensity physical activity, education level, income, smoking and menopausal status. Participants were also divided into sex-specific tertiles of baseline sedentary behaviour and change in sedentary behaviour from baseline to follow-up, and an ANCOVA was then used to compare the change in markers of adiposity across these tertiles, adjusting for the same covariates as in the above regression analyses. It should be noted that participants with identical values were grouped into the same tertile, which resulted in unequal numbers of participants in each tertile. A Bonferroni correction was used to adjust for multiple comparisons in post hoc tests following the ANCOVA.

Data are given as mean and standard deviation unless otherwise noted. Statistical significance was set at a *p* value of <0.05. All statistical analyses were performed using SAS version 9.2 (SAS Institute, Cary, NC).

## Results

### Baseline Sedentary Behaviour and Markers of Cardiometabolic Risk

Baseline characteristics of male and female participants are presented in Tables 1 and 2. At baseline, men and women averaged 8.3 and 7.5 hours of daily sedentary behaviour, respectively. Sedentary behaviour was not associated with any marker of adiposity or cardiometabolic risk in unadjusted cross-sectional analyses at baseline (Tables 3 and 4). These results were not changed following adjustment for age and sex. Following additional adjustment for energy intake, moderate-to-vigorous physical activity, educational level, income, smoking and menopausal status, each additional 15-minutes of baseline sedentary behaviour was cross-sectionally associated with 0.03 kg/m^2^ lower BMI (95% CI = −0.05, −0.01) in women, but not men. However, there were no other significant associations between sedentary behaviour and any other marker of adiposity or cardiometabolic risk in the fully adjusted model at baseline.

**Table 3:** Associations (95% confidence interval) of sedentary behaviour and markers of adiposity at baseline.

Model	BMI (M)	BMI (F)	WC	Fat%	TAAT	VAT	ASAT
1	−0.01 (−0.08, 0.07)	−0.02 (−0.12, 0.09)	0.07 (−0.10, 0.24)	−0.10 (−0.22, 0.01)	−1.51 (−4.09, 1.07)	−0.07 (−0.95, 0.81)	−1.45 (−3.43, 0.53)
2	−0.01 (−0.08, 0.07)	0.01 (−0.10, 0.11)	0.02 (−0.14, 0.17)	−0.01 (−0.10, 0.08)	−0.36 (−2.92, 2.21)	0.18 (−0.59, 0.94)	−0.54 (−2.51, 1.44)
3	−0.01 (−0.02, 0.01)	−0.03 (−0.05, −0.01)*	0.03 (−0.10, 0.16)	0.08 (−0.02, 0.17)	0.59 (−1.39, 2.57)	0.63 (−0.82, 2.08)	−0.04 (−1.55, 1.47)

Model 1: unadjusted.

Model 2: adjusted for age and sex.

Model 3: adjusted for age, sex, BMI, energy intake, moderate-to-vigorous physical activity, educational level, income, smoking and menopausal status.

* *p*<0.05.

M, Male; F, Female; BMI, Body Mass Index; WC, Waist Circumference; Fat%, body fat percentage; TAAT, total abdominal adipose tissue; VAT, visceral adipose tissue; ASAT, abdominal subcutaneous adipose tissue.

**Table 4:** Associations (95% confidence interval) of sedentary behaviour and markers of cardiometabolic risk at baseline.

Model	HDL-C (M)	HDL-C (F)	LDL-C	TG	FG	FI	HOMA-IR	Glucose AUC	Insulin AUC
1	−0.01 (−0.01, 0.01)	−0.01 (−0.01, 0.01)	−0.01 (−0.02, 0.01)	0.01 (−0.02, 0.03)	0.01 (−0.01, 0.01)	0.02 (−0.67, 0.71)	0.01 (−0.02, 0.03)	−0.21 (−3.27, 2.85)	88.64 (−660.03, 837.32)
2	−0.01 (−0.01, 0.01)	−0.01 (−0.01, 0.01)	−0.01 (−0.01, 0.01)	0.01 (−0.02, 0.03)	0.01 (−0.01, 0.01)	−0.11 (−0.83, 0.60)	−0.01 (−0.03, 0.03)	0.47 (−2.50, 3.44)	197.84 (−571.27, 966.94)
3	−0.01 (−0.01, 0.01)	0.01 (−0.01, 0.02)	0.01 (−0.02, 0.03)	−0.01 (−0.03, 0.01)	−0.01 (−0.02, 0.01)	−0.03 (−1.17, 1.11)	−0.01 (−0.06, 0.04)	−6.37 (−13.37, 0.62)	823.09 (−453.45, 2099.64)

Model 1: unadjusted.

Model 2: adjusted for age and sex.

Model 3: adjusted for age, sex, BMI, energy intake, moderate-to-vigorous physical activity, educational level, income, smoking and menopausal status.

* *p*<0.05.

M, Male; F, Female; HDL-C, HDL-Cholesterol; LDL-C, LDL-Cholesterol; TG, triglycerides; FG, fasting glucose; FI, fasting insulin; AUC, area under-the-curve.

Tables 5 and 6 present associations of baseline sedentary behaviour with changes in measures of adiposity and markers of cardiometabolic risk, respectively. In unadjusted analyses each 15-minute increase in baseline sedentary behaviour was associated with a 0.01 mmol/L increase in HDL-cholesterol (95% CI = 0.01, 0.01) and a −0.04 kg/m^2^ reduction in BMI (95% CI = −0.08, −0.01) in women, but not men. Following adjustment for age and sex, the association with BMI in women remained unchained, while each 15-minute increase in baseline sedentary behaviour was also associated with a 0.01 mmol/L reduction in LDL-cholesterol (95% CI = −0.01, −0.01), a 0.67 pmol/L increase in fasting insulin (95% CI = 0.10, 1.25), and a 0.03 unit increase in HOMA-IR (95% CI = 0.01, 0.05) in men and women combined. However, after further adjustment for baseline BMI, energy intake, moderate-to-vigorous physical activity, educational level, income, smoking and menopausal status, baseline sedentary behaviour was not associated with changes in any marker of adiposity or cardiometabolic risk.

**Table 5:** Associations (95% confidence interval) of baseline sedentary behaviour with 6-year change in markers of adiposity.

Model	BMI (M)	BMI (F)	WC	Fat%	TAAT	VAT	ASAT
1	0.01 (−0.02, 0.05)	−0.04(−0.08, −0.01)*	−0.02(−0.09, 0.05)	−0.03(−0.08, 0.02)	−0.47(−1.57, 0.63)	−0.23(−0.73, 0.26)	−0.24(−1.05, 0.58)
2	0.01(−0.02, 0.05)	−0.04(−0.08, −0.01)*	−0.02(−0.10, 0.05)	−0.03(−0.08, 0.02)	−0.33(−1.47, 0.81)	−0.19(−0.70, 0.33)	−0.14(−0.98, 0.70)
3	−0.02(−0.08, 0.05)	0.03(−0.05, 0.11)	−0.07(−0.19, 0.05)	−0.07(−0.15, 0.02)	−0.69(−2.92, 1.53)	−0.16(−1.38, 1.05)	−0.53(−1.91, 0.86)

Model 1: unadjusted.

Model 2: adjusted for age and sex.

Model 3: adjusted for age, sex, baseline BMI, energy intake, moderate-to-vigorous physical activity, educational level, income, smoking and menopausal status.

* *p*<0.05.

M, Male; F, Female; BMI, Body Mass Index; WC, Waist Circumference; Fat%, body fat percentage; TAAT, total abdominal adipose tissue; VAT, visceral adipose tissue; ASAT, abdominal subcutaneous adipose tissue.

**Table 6:** Associations (95% confidence interval) of baseline sedentary behaviour with 6-year change in markers of cardiometabolic risk.

Model	HDL-C (M)	HDL-C (F)	LDL-C	TG	FG	FI	HOMA-IR	Glucose AUC	Insulin AUC
1	−0.01 (−0.01, 0.01)	0.01 (0.01, 0.01)*	−0.01 (−0.01, 0.01)	−0.01 (−0.03, 0.02)	0.01 (−0.01, 0.01)	0.51 (−0.05, 1.08)	0.02 (−0.01, 0.05)	−0.58 (−4.07, 2.92)	−8.53 (−579.88, 562.82)
2	−0.01 (−0.01, 0.01)	0.01 (−0.01, 0.01)	−0.01 (−0.01, −0.01)*	−0.01 (−0.03, 0.02)	0.01 (−0.01, 0.01)	0.67 (0.10, 1.25)*	0.03 (0.01, 0.05)*	0.06 (−3.45, 3.57)	65.56 (−524.90, 656.02)
3	−0.01 (−0.01, 0.01)	0.01 (−0.01, 0.02)	−0.01 (−0.03, 0.01)	−0.01 (−0.02, 0.02)	−0.01 (−0.03, 0.01)	0.18 (−0.99, 1.35)	0.01 (−0.07, 0.07)	−2.63 (−12.51, 7.25)	−1252.36 (−2527.59, 22.86)

Model 1: unadjusted.

Model 2: adjusted for age and sex.

Model 3: adjusted for age, sex, baseline BMI, energy intake, moderate-to-vigorous physical activity, educational level, income, smoking and menopausal status.

* *p*<0.05.

M, Male; F, Female; HDL-C, HDL-Cholesterol; LDL-C, LDL-Cholesterol; TG, triglycerides; FG, fasting glucose; FI, fasting insulin; AUC, area under-the-curve.

**Figure 1:**
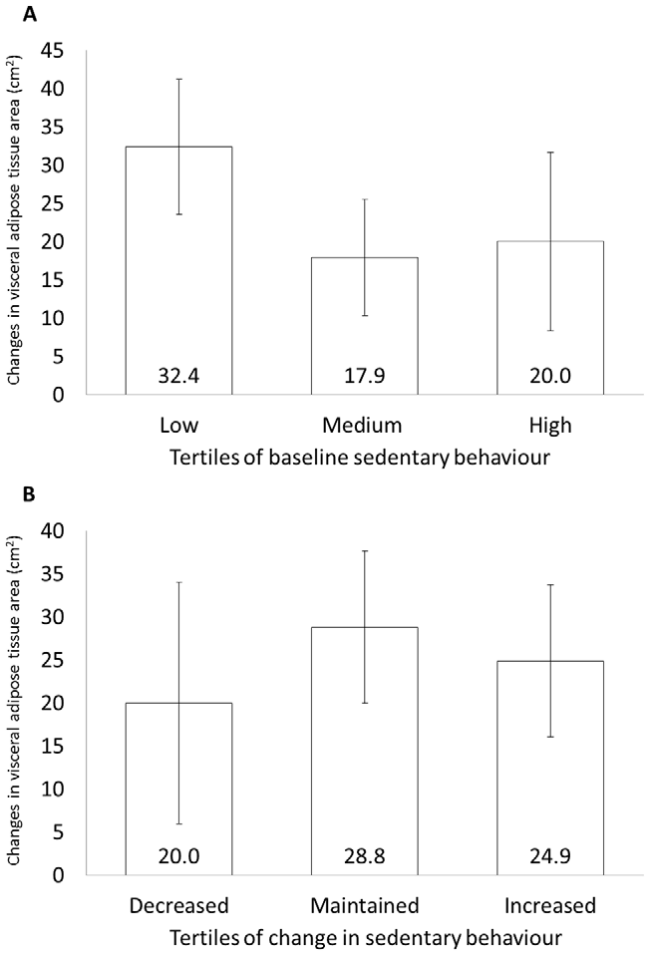
Changes in visceral adipose tissue across tertiles of sedentary behaviour. Changes in visceral adipose tissue cross-sectional area across tertiles of baseline sedentary behaviour (Figure 1A) or change in sedentary behaviour (Figure 1B) were compared by analysis of covariance with adjustment for age, sex, baseline BMI, energy intake, moderate-to-vigorous physical activity, educational level, income, smoking and menopausal status. Data are presented as mean ± standard error. There were no significant differences across tertiles of sedentary behaviour in either analysis.

Figure 1A presents the average accumulation of visceral adipose tissue across the three tertiles of baseline sedentary behaviour. The mean (standard deviation) reported sedentary time in the three tertiles were 305 (66), 471 (53) and 642 (89) minutes in the low, medium, and high tertiles, respectively. There were no differences in the accumulation of any abdominal fat compartment across the three tertiles of sedentary behaviour. Adjusting for covariates did not materially change the results.

### Longitudinal Changes in Sedentary Behaviour and Markers of Cardiometabolic Risk

In the fully adjusted model, each 15-minute increase in sedentary behaviour was positively associated with a 0.13 cm increase in waist circumference (95% CI = 0.02, 0.25). However, there were no significant associations between the change in sedentary behaviour and the change in visceral adiposity or any other marker of cardiometabolic risk (data not shown).

Participants were also classified into tertiles based on their longitudinal changes in sedentary behaviour from baseline to follow up. One third of participants reduced their sedentary time by a mean (standard deviation) of 195 (108) minutes over the 6-year follow-up. Another third maintained roughly the same amount of sedentary behaviour throughout the study, reducing their sedentary time by an average of just 13 (39) minutes. The final third of participants increased their sedentary time by an average of 165 (97) minutes. However, there were no differences in the accumulation of any abdominal fat compartment among these three tertiles (Figure 1B).

## Discussion

Our results suggest that sedentary behaviour is not associated with 6-year changes in visceral adiposity in adult men and women. To our knowledge, this is the first longitudinal study to examine the relationship between sedentary behaviour and the accumulation of visceral adipose tissue measured by CT. These findings are consistent with a recent study that found no cross-sectional association between objectively measured sedentary behaviour and visceral adiposity in a group of 126 abdominally obese men and women [12].

It is worth noting that both our study, and the previous study by McGuire and Ross [12], examined the association of visceral fat with a measure of total sedentary time. It is unclear whether similar results would have been observed for specific modalities of sedentary behaviour (e.g. screen-based vs. non-screen sedentary behaviours). For example, prospective studies in both the US and Australia have found associations between TV viewing and increased waist circumference [7], [8]. Given that TV viewing has been linked with increased energy intake [3], [28], [29] this modality of sedentary behaviour may be more closely associated with changes in adiposity and metabolic risk than global measures of total sedentary time [30]–[32]. For example, a study of 9,000 American adults found that those who watched more than 2 hours per day of television also consumed higher amounts of energy-dense snack foods and soft drinks, as well as consuming more calories during snacks and the evening meal [32]. Further, a recent intervention study by Harris et al. [33] reports that exposure to food advertisements resulted in roughly a 30% increase in food intake among adult participants. Other specific forms of sedentary behaviour such as seated mental work have also been shown to result in increased food intake, as compared to simply resting in the seated position [34]. Taken together, these findings suggest that specific modalities of sedentary behaviour are likely to impact food intake (and therefore adiposity) in different ways, and highlight the importance of assessing the impact of both global sedentary behaviour and of these specific modalities [35].

With the exception of waist circumference, the present study also failed to detect a prospective association between sedentary behaviour and several important markers of cardiometabolic risk including plasma lipids, HOMA-IR, and glucose tolerance. These findings are consistent with some, but not all, previous prospective studies in this area. For example, Ekelund and colleagues found no association between baseline sedentary behaviour and HOMA-IR at 1-year follow-up in a group of 192 men and women [36]. In contrast, Helmerhorst and colleagues reported a significant association between baseline sedentary behaviour and fasting insulin at 5-year follow-up in a cohort of 376 adults, independent of physical activity levels [37]. Of note, both of these studies used objective measures of total sedentary time at baseline. Wijndaele and colleagues have also reported prospective associations between changes in television viewing and clustered cardiometabolic risk in women, but not men [8].

As with adiposity, it is likely that the relationship between sedentary behaviour and markers of cardiometabolic risk may also vary depending on the modality of sedentary behaviour. For example, we have previously reported that seated video-game use, but not other forms of sedentary behaviour, are associated with increased metabolic risk in overweight and obese adolescents [38]. Given these and other findings, it is difficult to come to global conclusions regarding sedentary behaviour and the development of subsequent cardiometabolic risk. However, given the consistent associations between sedentary behaviour and the risk of mortality reported in other studies, public health messages promoting reductions in sedentary behaviour remain important [3].

The present study contains strengths and weaknesses that warrant mention. Limitations include the measurement of sedentary behaviour by self-report, and a lack of information related to specific modalities of sedentary behaviour. The observed results may have differed if an objective measure of sedentary behaviour had been employed, or if sedentary behaviour had been broken into specific modalities such as screen-time and non-screen sedentary behaviours. The relatively small sample size and homogeneous sample of the current study also limits our statistical power, and the generalizability of these findings. It should also be noted that this was a retrospective analysis, as the Quebec Family Study was originally designed to assess the genetic contributions to obesity. Strengths of this study include its longitudinal design and the use of computed tomography to assess visceral and subcutaneous abdominal adiposity [39]. This study also included objective measures of several important markers of cardiometabolic risk, including lipids, insulin resistance, and glucose tolerance in both men and women studied in their natural environment.

In summary, our results suggest that neither baseline sedentary behaviour nor changes in sedentary behaviour are associated with longitudinal changes in visceral adiposity in adult men and women. With the exception of waist circumference, sedentary behaviour does not appear to be associated with longitudinal changes in any marker of cardiometabolic risk in this population. These findings suggest that the development of cardiometabolic risk may be due primarily to factors other than self-reported sedentary behaviour.
